# Physical Activity Levels and Domains Assessed by Accelerometry in German Adolescents from GINIplus and LISAplus

**DOI:** 10.1371/journal.pone.0152217

**Published:** 2016-03-24

**Authors:** Maia P. Smith, Dietrich Berdel, Dennis Nowak, Joachim Heinrich, Holger Schulz

**Affiliations:** 1 Institute of Epidemiology I, Helmholtz Zentrum München – German Research Center for Environmental Health, Neuherberg/Munich, Germany; 2 Research Institute, Department of Pediatrics, Marien-Hospital Wesel, Wesel, Germany; 3 Comprehensive Pneumology Center Munich (CPC-M), Member of the German Center for Lung Research, Munich, Germany; 4 Institute and Outpatient Clinic for Occupational, Social and Environmental Medicine, Ludwig-Maximilians-University, Munich, Germany; Vanderbilt University, UNITED STATES

## Abstract

**Background:**

Physical activity (PA) is a well-known and underused protective factor for numerous health outcomes, and interventions are hampered by lack of objective data. We combined accelerometers with diaries to estimate the contributions to total activity from different domains throughout the day and week in adolescents.

**Methods:**

Accelerometric and diary data from 1403 adolescents (45% male, mean age 15.6 ± 0.5 years) were combined to evaluate daily levels and domains of sedentary, light, and moderate-to-vigorous activity (MVPA) during a typical week. Freedson’s cutoff points were applied to determine levels of activity. Total activity was broken down into school physical education (PE), school outside PE, transportation to school, sport, and other time.

**Results:**

About 2/3 of adolescents’ time was spent sedentary, 1/3 in light activity, and about 5% in MVPA. Boys and girls averaged 46 (SD 22) and 38 (23) minutes MVPA per day. Adolescents were most active during leisure sport, spending about 30% of it in MVPA, followed by PE (about 20%) transport to school (14%) and either school class time or other time (3%). PE provided 5% of total MVPA, while leisure sport provided 16% and transportation to school 8%. School was the most sedentary part of the day with over 75% of time outside PE spent sedentary.

**Conclusions:**

These German adolescents were typical of Europeans in showing low levels of physical activity, with significant contributions from leisure sport, transportation and school PE. Leisure sport was the most active part of the day, and participation did not vary significantly by sex, study center (region of Germany) or BMI. Transportation to school was frequent and thus accounted for a significant fraction of total MVPA. This indicates that even in a population with good access to dedicated sporting activities, frequent active transportation can add significantly to total MVPA.

## Background

Physical activity (PA) is gaining increased attention as a modifiable protective factor for most of the common diseases of the developed world, including cardiovascular disease and obesity [1, 2] as well as being associated with lower all-cause mortality [3]. The World Health Organization suggests that children and adolescents should get 60 minutes moderate-to-vigorous physical activity (MVPA) per day. [4] In German adolescents, PA behaviour has so far been mainly studied by questionnaires, e.g. in the KiGGS study [5–7] and only limited information is obtained by objective measures, e.g. in HELENA [8, 9]. While it is generally agreed that school-age children are insufficiently active, little is known of activity allocation throughout the day or the relative importance of different activity domains in determining the observed high levels of sedentary behaviour and low levels of moderate or vigorous activity. [10, 11]

Past studies have often relied on self-reports, or parental reports, to measure PA levels. These generally overestimate time in sport [12–14]; assume subjects are continuously active during sport, which they typically are not; [15–17] and underestimate or do not consider PA that takes place outside of dedicated activities. For all these reasons, the reported correlation between self-reported and objectively measured PA is weak [12] so objective measurements of PA are increasingly used in field studies.

Accelerometry is one of the most popular objective methods for assessing PA outside the laboratory. Accelerometric measures correlate well with metabolic measurements in controlled studies under laboratory conditions [14], and can also assess activity during daily or weekly routine under field conditions. Subjects wear a motion sensor typically for a few days or a week; sometimes diaries are used to connect the documented motion with specific activities such as school physical education, [15] sport [16], or recess [15]. Following data validation, various algorithms are available to convert accelerometric counts epoch-by-epoch (one minute being a typical epoch length for children and adolescents) into estimates of activity level during that epoch. Based on metabolic demands, these levels are *sedentary*, *light*, *moderate* and *vigorous activity*. Health recommendations for children and healthy adults generally focus on daily or weekly minutes of MVPA.

Our goals in this study are:

- To quantify the amounts of sedentary, light, moderate, and vigorous physical activity in a large cohort of German adolescents;
- To assess the relative importance of different activity domains (school physical education (PE), school outside PE, transportation to school, and leisure sport) in determining total objectively-measured MVPA and VPA.

## Methods

### Study population / Approach of participants

This study sampled adolescents from two different German birth cohorts: GINIplus and LISAplus, born between 1995 and 1999 in Munich or Wesel. Accelerometry was done between 2011 and 2014, and subjects were 15.6 (SD 0.5) years old at the time of accelerometry. GINIplus (German Infant Study on the influence of Nutrition Intervention plus environmental and genetic influences on allergy development) is an ongoing prospective cohort, initiated to investigate the influence on allergy development of nutrition intervention during infancy, air pollution and genetics. [18–20] LISAplus (Influence of Life-style related factors on the development of the Immune System and Allergies in East and West Germany, Plus the influence of traffic emissions and genetics) is an ongoing population-based birth cohort of unselected infants, designed to assess influence of lifestyle factors, traffic emissions and genetics on the development of the immune system and allergies in East and West Germany. Details on study design are published elsewhere. [18, 19, 21, 22]

**Fig 1:**
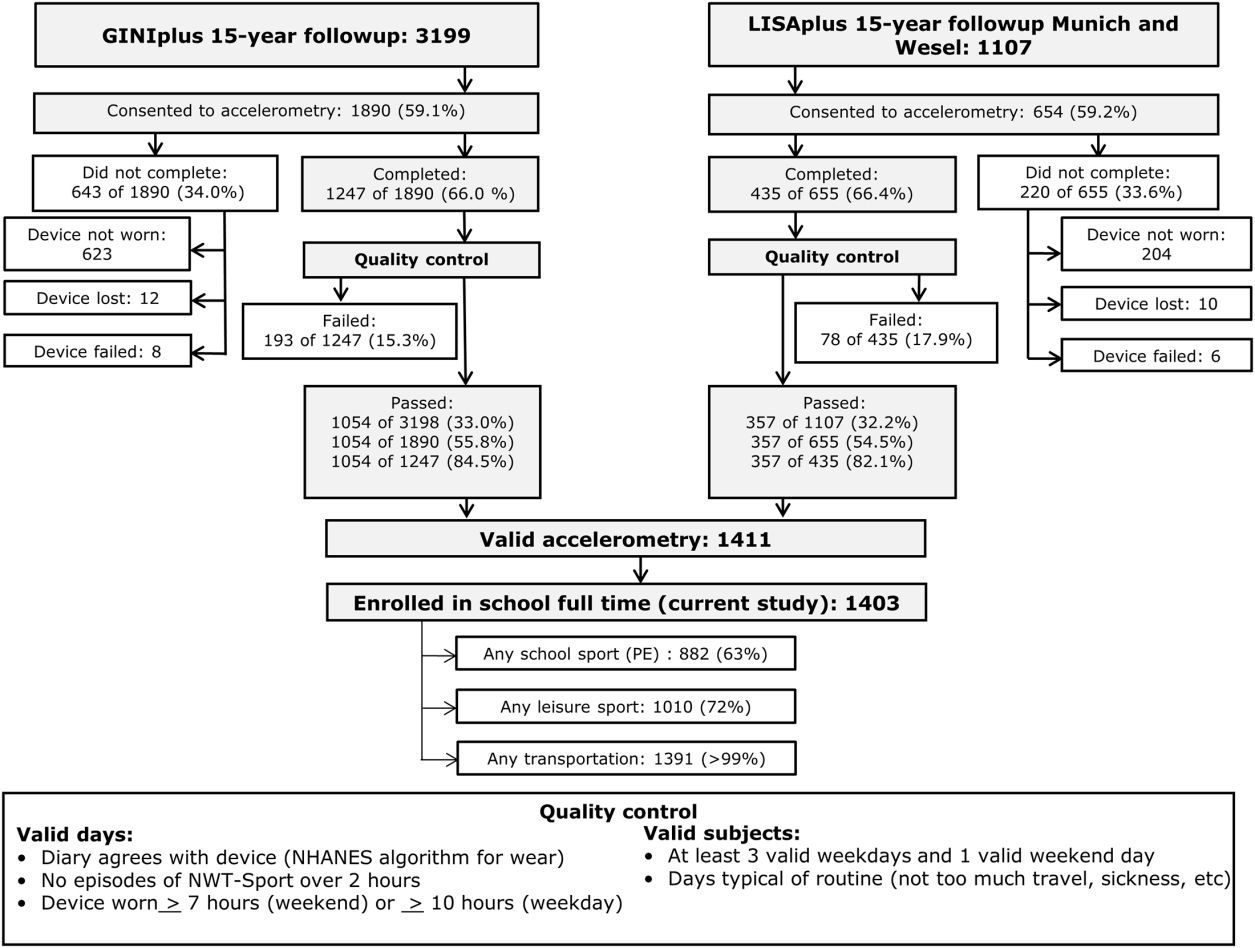
Accelerometry Response Rate. GINIplus15 and LISAPlus15 are the 15-year followups of birth cohorts GINIplus and LISAPlus. For details on GINIplus and LISAplus see von Berg et al, 2003 and 2015 [24–26]and Chen et al, 2007 [19] respectively. We give the number and percentage of subjects as a percentage of those contacted for accelerometry, those who gave their consent, and those who completed accelerometry and returned the device. Quality control failure includes inconsistency between diary and weartime criteria of Troiano et al, 2007 [27] using SAS programs published by NHANES [28](58% of excluded days); other non-wear time issues (27% of excluded days); and technical issues (7.4% of excluded days). Many days were invalid for more than one reason.

Accelerometry participants were recruited from the entire 15-year followup of GINIplus and LISAplus that resided in the study centers Munich and Wesel, which includes all of GINIplus but only 64% of LISAplus. For a flowchart see Fig 1; for more details on followup see [23]. Attempts were made to contact subjects at age 15 by paper mail, electronic mail, and/or telephone, and at this time consent for accelerometry was requested. If consent was given, subjects received a postcard one week before the scheduled start time of accelerometry requesting consent to send the device. Those who confirmed consent by postcard were sent the device. Almost all of these (>99%) returned the device after the scheduled week.

Of the 3199 subjects from GINIplus who were successfully recontacted at age 15, all were approached for accelerometry, 1890 (59%) gave initial consent and 1247 (66%) gave final consent, completed successfully, and returned the device. Of the 1740 subjects from LISAplus who were successfully recontacted, 1107 (64%) were from Munich or Wesel and thus approached for accelerometry. Of these 654 (59%) gave initial consent and 435 completed (66%). Of the 1682 adolescents from GINIplus and LISAplus who completed accelerometry, 1411 (83%) successfully passed data-quality checks. Of these, 1403 were enrolled in school full time. These 1403 subjects (8781 days) are profiled in the current study.

Recruitment, response, and exclusions of subjects are shown in Fig 1.

### Ethics, Consent and Permissions

Studies were conducted in Germany, in the Munich and Wesel area. The approval of the Ethics Committees includes the written consent procedure. Written informed consent was obtained from the parents or the legal guardian of all participants.

Initial recruitment of the GINI and LISA cohorts was approved by the Department of Medicine, of Ludwig Maximilians University, Munich (ethics reference number 111/94 (GINI) and 138/97 (LISA)); the Board of Physicians of West Rhine-Westphalia (reference number 9061 (GINI)) and the Medical Faculty of the University of Leipzig (reference number 560 (LISA)). The 15-year followups were approved by the respective local Ethics Committees, the Bavarian Board of Physicians (Bavarian General Medical Council reference number 10090 (GINI) and 12067 (LISA)); the Medical Council of North-Rhine-Westphalia (reference number 2102446 (LISA)); and the Board of Physicians in Saxony (reference number EK-BRO2 (LISA.)

### Accelerometry

Accelerometry protocol was the same as in [13] and [29]. Subjects who confirmed consent to accelerometry were mailed a package containing the following: two monitors including mounting strips with identification labels for hip and foot, personalized cover letter, brief instruction sheet, and pre-printed diary with instructions for filling it in and sample of proper methods [13], and a stamped, pre-addressed envelope for returning monitors after one week of PA measurement. Almost all devices were successfully returned.

Triaxial accelerometers (ActiGraph GT3X, Pensacola, Florida) were worn on the dominant hip for 7 consecutive days, after which they were returned by mail. This device has demonstrated validity in adolescents [30, 31] and performed satisfactorily in our sample: less than 2% of accelerometers (see Fig 1) were either lost or suffered technical failure as detected by flashing light or implausibly continuous high or low counts as compared with the diary. While this device is not waterproof, the 14 technical failures include any damage due to water exposure, such as that caused by failure to remove the device during swimming or showering.

#### Data Management and Quality Control

Sampling rate was set to 30 Hz and the measured accelerations stored at 1 Hz after conversion into activity counts. Counts were summed over 60-second epochs. This was the most common length in two recent reviews: Cain et al [4], found that of 68 accelerometric studies in adolescents, 63% used an epoch length of 60 seconds, 13% used 30 seconds, 4.4% 15 seconds and 3% less than 5 seconds, and Guinhoya et al [11] found that 60-second epochs were the commonest even when including studies of children, as well as adolescents. Furthermore, it is known from sport physiology [32] that cardiovascular adaptation to a certain exercise level takes about 1 to 2 minutes before reaching a steady state. Thus the physiological benefits of very brief epochs of PA have not been ascertained, and our choice of 60 seconds represents both clinical relevance and maximal intercomparability with other studies.

Data filtering was set to default (‘normal’) as recommended by ActiGraph. Activity counts of all three axes (vertical, horizontal and mediolateral), the inclinometer signal, and number of steps were measured. ActiLife software was used for initialization of accelerometers (version 5.5.5, firmware 4.4.0) and for download of data. Activity counts were assigned to the four intensity levels—sedentary, light, moderate, and vigorous physical activity—using Freedson’s et al (2005) commonly-used uniaxial cut-points for children [33]. PA data were checked to identify invalid days both by visual inspection and by semiautomatic methods.

#### Activity Diary

Subjects were instructed to document each of the following events as close as possible to the time they occurred: time of waking up and going to bed; time and reason for removing one or both monitors (non-wear time, NWT) such as for showering or swimming; time and method of travel to and from school, such as by walking or driving; time of starting and finishing school; time of starting and finishing school sport; and time and type of sporting activity. A note at the end of the 7^th^ recording day instructed the participant to stop the measurement and immediately return monitors and diary to the study center in the provided envelope. [13]

Diary information was digitized using a 7-day template and a specific coding for events such as sickness, trips, type of sport performed, and NWT. Data entries were reviewed by a second study assistant to avoid transcription errors. Since the goal of this study was to capture a representative sample of daily PA, days were disqualified if they were judged to be not representative of typical routine as described in Pfitzner et al [13]. Diary-based reasons for exclusion of a day were: traveling for more than one day or reported common cold or headache for more than one day (excluded from the second day on). Days were excluded if the sensor was worn incorrectly or the subject reported flashing LED indicative of technical problems.

#### Validation of wear time

NWT was identified both by visual inspection of accelerometer tracings and by comparing the diary data to the results from the monitor according to the NWT algorithm of Troiano et al [27] using SAS programs published by NHANES.[34] These programs identify probable NWT as at least 60 minutes of consecutive zero counts with less than two consecutive intervals with counts less than or equal to 100. In most cases the diary agreed with the automatic programs upon wear time and NWT. In the event of large discrepancies between the NHANES criteria and the diary entry, the measuring day was excluded from analysis.

A large discrepancy was defined as being more than 45 minutes of diaried NWT when the program indicated the device was worn, or more than 150 minutes where the program indicated NWT but the diary did not. These limits are the 10^th^ and 90^th^ percentiles for daily discrepancy in a subset of this cohort, [13] following the recommendation [35] to specify the accepted level of uncertainty in PA data. Days with smaller discrepancies were included in the analysis, with activity domain and weartime determined from the diary entries.

Of a total 11,572 recorded days, 2740 (17.1%) were invalid. Most invalid days (1140, 58%) were the result of inconsistency between the diary and the NHANES weartime criteria, reflecting our high standard of data cleaning and suggesting a relatively accurate allocation of activity on the days that passed quality control. Other reasons included non-wear time issues (526 days, 26.7%), and technical issues (145 days, 7.4%). Many days were invalid for more than one reason. In addition, 271 days were excluded because the subject did not have at least 3 valid weekdays and one valid weekend day.

#### Handling of non-wear time during sport

When the accelerometer was removed during -time sport or school PE (in the diary as NWT-Sport or NWT-PE) it was necessary to account for the missing time in order to avoid underestimating total PA levels. NWT-Sport (or PE) was rare, occurring on only 297 / 8781 valid days (3.4%).

For periods of documented NWT- Sport longer than 30 minutes but shorter than 120 minutes, 15 minutes was subtracted before imputation in order to account for non-sport activities such as showering and changing clothes. For longer periods of NWT-Sport, e.g. during a competition, imputation would become unreliable, so any single period of NWT-Sport (either or PE) longer than 120 minutes disqualified that day from inclusion. When imputing activity, school PE and sport were kept separate; imputation was not used for any other periods of NWT. For each domain, imputation was done by dividing total moderate and vigorous PA from that domain, with total device weartime during that domain; assuming that those average levels were maintained during NWT-Sport or NWT-PE; and imputing the missing time with average levels. If the subject had any periods of that activity domain (sport or PE) where the device was worn, his own average was used; if not then sex-specific averages from the whole study population were used. Imputation increased average MVPA by about a minute per day (1.1 minutes for boys, 0.54 for girls.)

#### Validation of days

Since our goal was to measure typical activity, subjects were required to have at least one valid weekend day of recording in addition to at least three valid weekdays. This reflects the known differences in activity between weekends and weekdays and thus improves representativeness of data.

Days were required to have at least 10 hours of valid recording time to be considered valid, or as little as 7 hours if subjects were awake for less than 10 hours, as is recommended in [13] and [29]. This is not uncommon in adolescents, especially on weekends, and further reflects our desire to capture typical routine. Of 8781 days, only 116 (1.3%) had less than 10 hours recording.

### Activity domains

Every minute of accelerometer weartime fell into one of five categories (domains).

- **School physical education (PE) (882 subjects, 63%).** Time between diaried start and end of school physical education (PE), when both start and end were diaried. Since many German schools require PE, it is likely that most children who did not report any during valid accelerometric days still had it on other days.
- **School outside PE (1400 subjects, > 99%).** Time between diaried start and end of school, when subject was not in diaried school physical education (PE). The remaining 3 subjects attended school but did not wear the device during it. However, each still accumulated at least three valid weekdays (10 hours wear) and one weekend day.
- **Leisure sport (1010 subjects, 72%).** Time between diaried start and end of sport that took place outside school PE. This includes any activity the subject deliberately classifies as sport and reports in the diary, including both team sports such as football and individual sports such as jogging. Leisure sport in a subset of this cohort has been previously profiled. [29]
- **Transportation to school (1391 subjects, > 99%).** Time spent between diaried departure for school and arrival at school. The trip home from school sometimes took much longer than the trip to school (as much as 493 minutes) implying that subjects did not go straight home. As a result only the trip to school is described.
- **Other time.** All other waking time.

Data were not collected on any activities besides sleeping, NWT, and the stated activity domains.

### Collection and Definition of Confounders

In addition to the measured confounders age, height, weight, BMI, and BMI category we also considered socioeconomic status and study center (location) as possible correlates of PA. Confounders were defined as follows:

- **Height, weight, BMI and BMI category.** Height and weight were measured objectively at a physical exam at age 15. BMI was calculated from height and weight as kg/m^2^, and BMI category was taken from age- and sex- specific percentiles from a German reference population, [36] with 10^th^, 90^th^, and 97^th^ percentile defining the boundaries between underweight, normal weight, overweight, and obese.
- **Study center Munich compared with Wesel.** Subjects were drawn in roughly equal numbers from the urban region Munich, and the rural/suburban region of Wesel. These two cities may differ in the availability of active transportation, encouragement of PA, or other factors with potential to influence PA levels. Thus we checked whether study center was associated with leisure-sport and PE participation.
- **Parental education (socioeconomic status).** Socioeconomic status (SES) may be associated with PA through availability of leisure sport, neighbourhood characteristics affecting active commuting to school, cultural encouragement of PA, or other factors. High SES was defined as yes if the child’s better-educated parent reported entering university or higher, and no otherwise.

Selection bias was quantified by comparing anthropometric and sociodemographic characteristics of successful accelerometry completers, with the populations from which they were recruited, in Table 1.

**Table 1:** Population Characteristics/ Selection Bias within GINIplus and LISAplus.

	Entire cohort		Initial consent		Accelerometry completers		Study population			
	GINILISA 15-year followup Munich and Wesel		Gave initial consent to accelerometry		Completed accelerometry, returned the device		Passed quality control			
	Boys	Girls	Boys	Girls	Boys	Girls	Boys	Girls	Boys	Girls
**N**	4306		2544		1682		1403		**	
**Male, %**	51.1		48.9		47.4		46.3		<0.0001	
**Height, cm**	177 (7.5)	167 (6.3)	176 (7.5)	167 (6.2)	177 (7.6)	167 (6.2)	177 (7.4)	168 (6.2)	—	0.01
**Weight, kg**	65.1 (13)	58.7 (9.8)	65.1 (13)	58.8 (9.8)	64.8 (12)	58.8 (9.8)	64.5 (12)	59.0 (9.6)	—	—
**BMI, kg/m**^**2**^	20.8 (3.4)	21.0 (3.1)	20.8 (3.3)	21.0 (3.0)	20.7 (3.2)	20.9 (3.0)	20.6 (3.2)	21.0 (2.9)	—	—
**Study center Munich, %**	59	59	63	60	64	58	65	58	0.0001	—
**Parents highly educated**^2^**, %**	65	68	64	66	70	70	71	71	0.0004	0.06
**BMI category,**^3^**%**									0.03	—
**Underweight**	7.9	6.6	7.9	6.6	7.6	5.5	9.1	6.3	**	**
**Normal weight**	80	84	80	83	80	85	80	85	**	**
**Overweight**	7.9	6.1	7.9	6.4	8.7	5.9	8.6	5.0	**	**
**Obese**	4.1	3.6	4.0	3.7	3.3	3.9	2.7	3.9	**	**

Mean (SD) if not otherwise stated.

Values given as percent for binary and categorical variables, mean (median); 5^th^, 95^th^ percentiles for skewed, mean (SD) for centrally distributed.

^1)^ P-values for selection (study population vs. entire 15-year followup) given as Wilcoxon’s two-tailed rank-sum test for binary and otherwise noncentral variables, t-test for height and weight, Kruskal-Wallis for BMI category.—if p>0.10, ** if pairwise comparison inappropriate (see global null).

^2)^ If higher-educated parent entered university

^3)^ From age- and sex-specific 10^th^, 90^th^, and 97^th^ BMI cutpoints from a German reference population in (Kromeyer-Hauschild, 2001). P-value calculated for global null hypothesis (all categories equal) from Kruskal-Wallis test.

Group differences within the accelerometry cohort were quantified by comparing average daily activity (total time awake, accelerometric weartime, minutes in sedentary, light, moderate, vigorous, and MVPA) for the whole cohort and for the subsets that had any leisure sport and any PE. We also calculated the percentage of days each group achieved the 60 minutes MVPA recommended by the World Health Organization [4]. (Table 2).

**Table 2:** Population Characteristics and Selection.

	Study population (N = 1403)			Population with diaried leisure sport (N = 1010)				Population with diaried school sport (PE) (N = 882)			
	Boys	Girls	P for sex difference	Boys	Girls	P for sex-stratified difference^1^		Boys	Girls	P for sex-stratified difference^1^	
**Male (N, %)**	650 (46)		**	455 (45)		—		407 (46)		—	
**Age, years**	15.6 (0.5)	15.6 (0.5)	—	15.6 (0.5)	15.6 (0.5)	—	—	15.6 (0.5)	15.6 (0.5)	—	—
**Height, cm**	177 (7.4)	168 (6.2)	<0.0001	177 (7.4)	168 (6.2)	—	—	177 (7.4)	168 (6.3)	—	—
**Weight, kg**	64.5 (12)	59.0 (9.6)	<0.0001	64.0 (11)	59.0 (9.4)	—	—	64.4 (12)	58.5 (9.3)	—	0.09
**BMI, kg/m**^**2**^	20.6 (3.2)	21.0 (2.9)	0.001	20.4 (2.9)	21.0 (2.9)	0.04	—	20.6 (3.1)	20.8 (2.9)	—	0.1
**Study center Munich, %**	64.9	57.5	0.005	66.4	56.4	—	—	62.6	56.6	—	—
**Higher-educated parent entered university, %**	71.1	71.1	—	75.8	73.2	0.0001	0.04	70.9	71.2	—	—
**BMI category,**^4^**%**	**	**	**	**	**	**	**	**	**	**	**
**Underweight**	9.06	6.29	**	9.07	5.26	—	—	9.41	7.43	—	0.1
**Normal weight**	79.7	84.9	**	82.1	86.4	—	—	78.9	84.9	—	—
**Overweight**	8.59	4.95	**	7.03	5.08	—	—	9.41	4.25	—	—
**Obese**	2.70	3.88	**	1.81	3.27	—	—	2.29	3.40	—	—
**Any diaried leisure sport (%)**	70.0	73.7	—	**	**	**	**	74.2	76.4	0.003	0.03
**Any school physical education PE (%)**	62.6	63.1	—	66.4	65.4	0.003	0.03	**	**	**	**
**Accelerometric data, min/day;** Mean (SD)											
Time from getting up to going to bed (includes accelerometer non-wear, NWT) Mean (SD)	904 (52)	898 (49)	0.02	906 (50)	899 (49)			904 (52)	899 (47)		
**Accelerometer wear;**^3^^**,**^ ^4^ Does not include NWT Mean (SD)	887 (54	881 (49) 799, 962	0.01	888 (54)	881 (49)	—	—	887 (53)	882 (47)	—	—
**Sedentary**^3^	583 (79)	598 (69)	0.0001	580 (74)	594 (65)	—	0.005	583 (77)	597 (67)	—	—
**Light**^3^	260 (59)	246 (50)	<0.0001	261 (57)	250 (49)	—	0.0002	260 (60)	248 (49)	—	—
**Moderate**^3^^**,**^ ^4^	31.3 (14)	26.5 (15)	<0.0001	33.1 (14)	26.8 (14)	<0.0001	0.00035	32.2 (14)	26.9 (14)	0.049	0.02
**Vigorous**^3^^**,**^ ^4^	14.2 (12)	11.1 (11)	**	15.7 (12)	11.8 (11)	<0.0001	<0.0001	14.4 (12)	11.5 (10)	—	0.006
**MVPA**^3^^**,**^ ^4^	45.5 (22)	37.6 (23)	**	48.8 (22)	38.6 (20)	<0.0001	<0.0001	46.5 (23)	38.3 (22)	—	0.007
**MVPA difference from average**^3^^,^ ^4^	**	**	**	3.24	1.01	**	**	0.94	0.72	**	**
**% days with MVPA over 60 minutes**^3^^,^ ^4^	27.2	17.7	<0.0001	31.2	19.0	<0.0001	<0.0001	28.6	19.1	0.10	0.004
**MVPA over 60 minutes every day;** N, % subjects^3^^,^ ^4^	6, 0.92	8, 1.06	-	4, 0.88	5, 0.90	—	—	4, 0.98	6, 1.26	—	—

Mean (SD) unless otherwise stated.

Table 2 footer: P-values calculated using t-test for centrally distributed variables (age, height, BMI, weartime, sedentary, and light); Kruskal-Wallis for categorical (BMI category); Wilcoxon’s two-tailed rank-sum for binary and skewed (all others.)—if p>0.10, ** if pairwise comparison inappropriate (see global null.) For time and activity levels spent in various activity domains (leisure sport, school PE, transportation, school) see Table 4.

^1)^ P-value for difference from whole cohort

^2)^ From BMI cutpoints in (Kromeyer-Hauschild, 2001).[36]

^3)^ Freedson’s uniaxial algorithm for children from (Freedson, 2005) [34]as cited in (Trost, 2010).[34, 37]

^4)^ Data include estimated moderate and vigorous activity during nonwear time if the subject diaried they were in school PE or leisure sport.

Activity levels within each domain (total minutes, and minutes in sedentary, light, moderate, and vigorous activity) were quantified first only on days with that domain (Table 3), and then averaged over the week by domain (Table 4). Thus Table 3 shows how subjects allocated the time they spent in each activity domain, while Table 4 shows the relative importance of the five activity domains in determining total PA levels within this population.

**Table 3:** Profiles of Activity Domains. Domains profiled only on days when the subject reported the activity.

	School outside PE		PE^1^ (physical education)		Transport to school		Leisure sport^1^		All other waking time^1^	
	Boys	Girls	Boys	Girls	Boys	Girls	Boys	Girls	Boys	Girls
**Number of subjects with any (N (% of total)**	649 (>99)	751 (>99)	407 (63)	475 (63)	642 (99)	749 (99)	455 (70)	555 (74)	650 (100)	753 (100)
**Number of days with that domain, among subjects with any**	4.20 (0.92)	4.20 (0.94)	1.14 (0.40)	1.11 (0.33)	4.17 (0.95)	4.19 (0.95)	2.18 (1.3)	2.23 (1.3)	6.25 (0.86)	6.27 (0.88)
5^th^, 95^th^ percentile	3, 5	2, 5	1, 2	1, 2	2, 5	2, 5	1, 5	1, 5	5, 7	5, 7
**Total time** (min/day; mean (SD))(min / day; mean (SD)	333 (50)	336 (52)	84.1 (20)	82.2 (23)	33.1 (18)	35.5 (18)	111 (75)	95.2 (53)	609 (79)	600 (73)
**Sedentary**	241 (46)	266 (47)	19.2 (19)	22.2 (16)	15.5 (13)	18.2 (13)	23.3 (42)	20.9 (23)	403 (75)	399 (68)
**Light**	79.9 (32)	60.5 (25)	36.5 (19)	43.7 (19)	12.8 (5.9)	12.5 (5.6)	43.9 (45)	44.0 (31)	183 (50)	180 (43)
**Moderate**^1^	9.82 (6.6)	7.40 (5.8)	14.8 (8.9)	9.07 (6.6)	3.31 (2.9)	3.54 (3.2)	18.9 (14)	12.2 (14)	16.1 (9.8)	14.9 (11)
**Vigorous**^1^	2.28 (3.4)	1.32 (3.2)	9.27 (9.6)	4.34 (5.9)	1.47 (2.6)	1.28 (2.5)	14.4 (14)	10.5 (11)	6.85 (7.0)	5.86 (7.9)
**MVPA**^1^	12.1 (8.9)	8.72 (8.4)	24.0 (15)	13.4 (9.9)	4.78 (4.4)	4.82 (4.6)	33.3 (24)	22.7 (19)	23.0 (15)	20.7 (17)
**Activity during that domain: percent of timeof time**										
**Sedentary**	72.4	79.2	22.8	27.0	46.8	51.3	21.0	22.0	66.2	66.5
**Light**	24.0	18.0	43.4	53.2	38.7	35.2	39.6	46.2	30.1	30.0
**Moderate**	2.95	2.20	17.6	11.0	10.0	10.0	17.0	12.8	2.64	2.48
**Vigorous**	0.68	0.39	11.0	5.28	4.44	3.61	13.0	11.0	1.12	0.98
**MVPA**	3.63	2.60	28.5	16.3	14.4	13.6	30.0	23.8	3.78	3.45

See methods for domain descriptions and definitions.

^1)^Data include estimated moderate, vigorous and MVPA during diaried nonwear time due to school physical education (PE) or leisure sport when the device was not worn such as swimming (see methods). Sedentary and light activity are not imputed. Calculated with Freedson’s uniaxial algorithm for children from (Freedson, 2005) [33]as cited in (Trost, 2010).[33, 37].

**Table 4:** Activity Allocation by Domain, All Days.

	School outside PE		PE^1^ (physical education) (physical education)		Transport to school		Leisure sport^1^		Other time^1^	
	Boys	Girls	Boys	Girls	Boys	Girls	Boys	Girls	Boys	Girls
**Number of days (Mean** number of days each subject had that domain, including those with none)	6.24	6.27	0.71	0.70	4.11	4.16	1.52	1.64	6.24	6.27
5^th^, 95^th^ percentiles	5, 7	5, 7	0, 2	0, 2	2, 5	2, 5	0, 4	0, 5	5, 7	5, 7
Daily minutes within that domain **(Mean)**										
**Total**	224	225	9.23	9.01	21.9	23.7	27.3	25.7	609	600
Sedentary	163	179	2.15	2.43	10.3	12.2	5.41	5.60	403	399
Light activity	53.8	40.6	4.00	4.74	8.52	8.30	10.7	11.6	183	180
Moderate activity	6.60	4.90	1.61	1.00	2.18	2.34	4.81	3.39	16.1	14.9
Vigorous activity	1.53	0.89	1.02	0.49	0.97	0.85	3.86	3.03	6.85	5.86
MVPA	8.13	5.79	2.62	1.48	3.14	3.19	8.67	6.43	23.0	20.7
**Percent of total MVPA** (Mean)	19.2 3.65, 42.6	16.2	6.30	4.49	7.28	8.67	16.7	16.0	50.5	54.6
(5^th^, 95^th^ percentiles)	3.65, 42.6	2.73, 37.9	0, 19.2	0, 15.3	0.41, 20.7	0.47, 22.8	0, 54.9	0, 52.6	20.8, 82.7	23.7, 83.8
**Percent of total vigorous PA (Mean)**	12.0	7.34	9.36	6.35	5.93	6.28	22.4	24.4	50.3	55.6
(5^th^, 95^th^ percentiles)	0, 44.4	0, 32.1	0, 41.7	0, 27.5	0, 24.7	0, 27.1	0, 76.2	0, 79.4	10.8, 0.95	10.3, 100

Values include all subjects and all days, both with and without that activity domain.

Includes estimated moderate, vigorous and MVPA during diaried nonwear time due to school physical education (PE) or leisure sport when the device was not worn such as swimming (see Methods) Sedentary and light activity are not imputed. Activity levels calculated using Freedson’s uniaxial algorithm for children (Freedson, 2005) [33]as cited in (Trost, 2010).[37]. All analyses are averaged first by subject, then by day, in order to weight all subjects equally.

### Statistical Methods

All analyses were performed using SAS 9.2 or 9.3 (Cary, NC.) Intergroup comparisons were performed using t-test for height and weight; Kruskal-Wallis test for global null hypothesis for categorical values (BMI category) and Wilcoxon’s two-tailed rank-sum test for binary or nonnormal continuous variables. P-value for significance was 0.05, but p-values in the tables are shown up to 0.10.

## Results

### Population

Of the 4306 adolescents in the GINIplus and LISAplus 15-year followups (Table 1) 2544 gave initial consent to accelerometry, 1682 completed accelerometry, and 1403 (successful completers) passed quality control. Selection bias was evident in both consent to and completion of accelerometry: in particular completers were likelier to come from highly-educated families and to be female. Small differences were also apparent for study center Munich (completers tended to be urban) and for slightly greater height in girls.

### Daily Activity

PA was monitored in 1403 subjects for an average of 884 minutes (14.7 hours) per day and on average just over 6 days per subject, ranging from 4 to 7. (Table 2) Boys and girls averaged 45.5 (SD 22) and 37.6 (SD 23) minutes MVPA per day, and achieved over 60 minutes MVPA on 27% and 18% of days respectively. 1% of subjects (6 boys, 8 girls) achieved 60 minutes of MVPA every day. Subjects spent roughly 2/3 of time sedentary, 1/3 in light activity, and less than 5% in MVPA.

Adolescents with diaried leisure sport (N = 1010) or PE (N = 882) were comparable to the rest of successful accelerometry completers in both sociodemographic and anthropometric characteristics (Table 2). Averaged across all days, boys and girls with diaried leisure sport averaged 3.3 and 1.0 additional daily minutes of MVPA than the full population, and increased the fraction of days with over 60 minutes MVPA slightly from 27.2 to 31.2% (boys) and 17.7 to 19.0% (girls), both p <0.0001. (Table 2). Differences were smaller for PE, which was associated with 1.0 and 0.7 additional minutes MVPA per day (not significant for boys, p = 0.007 for girls) and one percentage point increase in days achieving the 60-minute recommendation (not significant for boys, p = 0.004 for girls).

### Profiles of Activity Domains

Participation in leisure-sport and PE was similar between the sexes. Adolescents with any leisure sport averaged 2.2 days with leisure sport, ranging from 1–5 days (5^th^ and 95^th^ percentiles, Table 3). On each day with leisure sport, boys and girls dedicated an average of 111 and 95.2 minutes to it (1.9 and 1.6 hours), getting 33.3 and 22.7 minutes MVPA from it (30 and 24% of sporting time.)

Boys and girls with any PE during valid recording, both averaged 1.1 days with PE over the course of the week (5^th^ and 95^th^ percentiles 1–2 days (Table 3)). On each day with PE, adolescents of both sexes spent about 83 minutes in PE; boys and girls got 24.0 and 13.4 minutes MVPA from it (28.5 and 16.3% of time). Lastly, on days with any transportation to school boys and girls spent 33.1 and 35.5 minutes in it, for a total of 4.8 minutes MVPA, or 14% of transportation time in MVPA.

The fraction of time spent in MVPA was comparable and low between school outside PE and other time, being 3.6 and 3.8% (boys) or 2.6 and 3.5% (girls). However, subjects were significantly more sedentary in school than outside it, spending 72 and 80% of school outside PE in sedentary behaviour compared with 66 and 67% of other time.

### Allocation of Total MVPA by Domain

This cohort dedicated almost an hour per day to domains that are expected to contribute significantly to MVPA: on an average day 9 minutes were spent in PE, 26 minutes in leisure sport, and 23 minutes in transportation to school (Table 4.) These domains made up only 31% of total MVPA, for an average of 12.7 minutes per day. Boys and girls averaged 17 and 16% of total MVPA from leisure sport, 7.3 and 8.7% from transport to school and 6.3 and 4.5% for PE, while school outside PE provided 19 and 16%. Finally, boys and girls accumulated 51 and 55% of total MVPA during other time.

While sporting activities made up only a small fraction of total MVPA, they contributed more significantly to vigorous PA (VPA.) The combination of PE and leisure sport accounted for only 21.7% of total MVPA, but 31.2% of VPA. (Table 4.)

**Fig 2:**
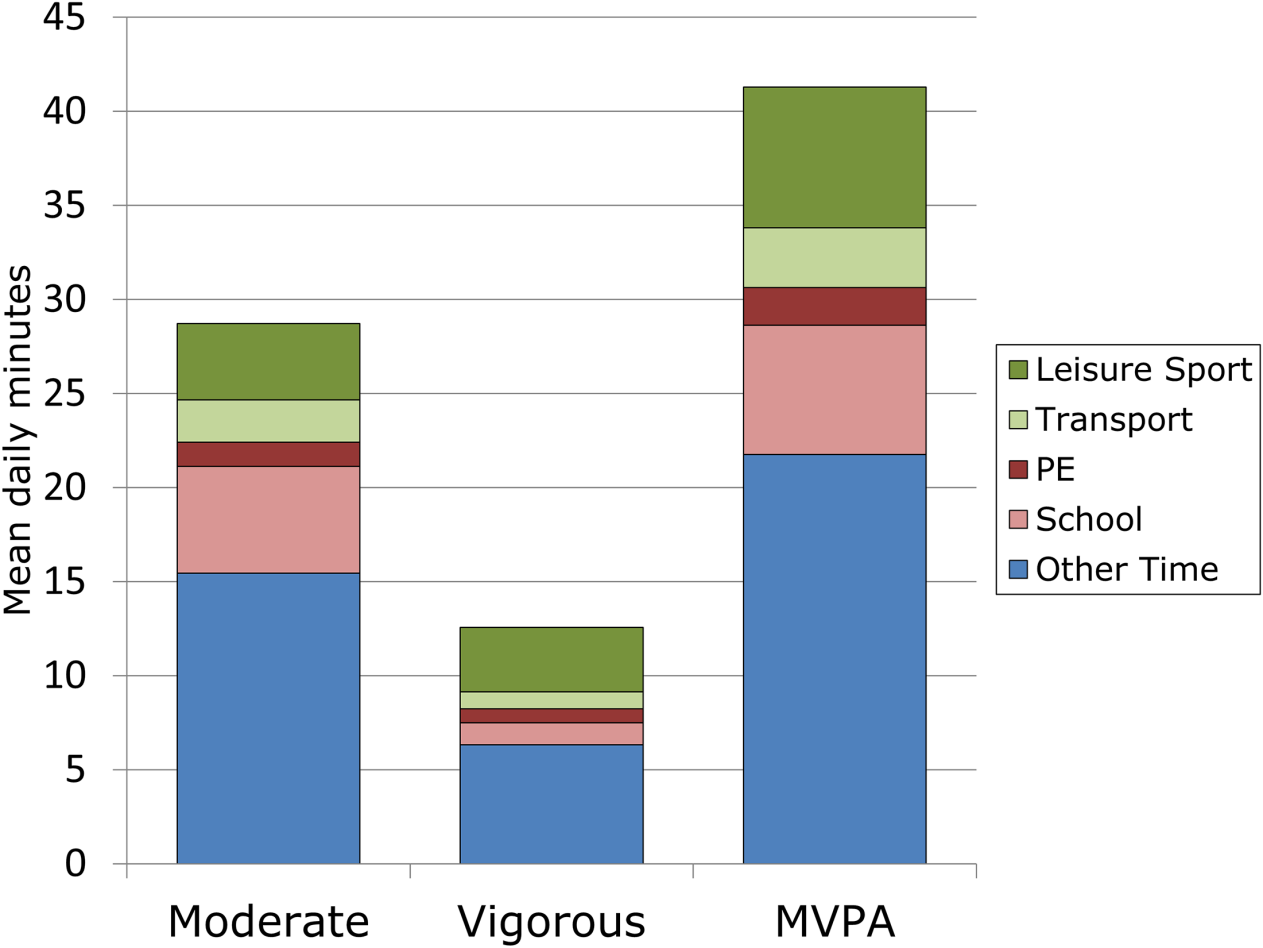
Daily Activity in German Adolescents. PE is any time between diaried start and end of school physical education (PE.) School is any time between diaried start and end of school, outside PE. Leisure Sport is any time outside of PE when the subject diaries sport. Transport is time between leaving home and arriving at school. Only the trip to school is described. Other is all other waking time when the monitor was worn. Estimates for PE and Leisure Sport include estimated moderate and vigorous activity during this period if the diary shows the device was not worn due to sport such as swimming. Calculations are based on data applying Freedson‘s vertical-axis algorithm for children from Freedson et al, 2005 [33] as cited in [37]. MVPA is moderate + vigorous physical activity.

Contributions of the five activity domains to total moderate, vigorous, and MVPA are profiled in Fig 2.

## Discussion

This is among the largest accelerometric studies of adolescents and, we believe, the first focusing on Germans specifically. In it we found the accelerometric PA levels of this cohort of German adolescents were below WHO recommendations [38]and comparable to, or even less than, estimates for either other European adolescents [9, 11] or younger American children. [39] [40] Boys were more active than girls, but were equally likely to participate in each activity domain that we considered. Adolescents got the recommended 60 minutes of MVPA on about a quarter of days, and almost no one achieved 60 minutes every day. Objective measurements also allowed us to precisely estimate sedentary time and light activity: the majority of time was spent sedentary, with most of the remainder in light activity and about 5% in MVPA.

Access to dedicated sporting time is high in this cohort, with most subjects choosing to participate in leisure sport as well as often having school sport (PE.) Adolescents of our cohort spent 15 and 30% of sporting time in MVPA, with less in PE than in leisure sport. This is less than others have found for leisure sport [16, 17, 41] but within the typical pre-intervention range observed for PE [42] particularly in older adolescents.

Subjects devoted comparatively little time to the domains that we had expected would contribute significantly to MVPA. The average day in our sample contained 9 minutes of PE, 26 minutes of sport, and 23 minutes of transportation to school. These domains accounted for only 31% of total MVPA, or 12.7 minutes per day ((2.0, 7.5 and 3.2 minutes, respectively).

Although little of the time spent in transportation to school was spent in MVPA, it was still frequent and long enough to make up a significant contribution to total MVPA. This underlines the potentially significant contribution of transportation to activity in adolescents, as has been found elsewhere. [43] Estimates of its contribution would be higher still if we had been able to include the trip home, since young Europeans often bicycle or walk to school. [43] Since almost all subjects devoted time to transportation anyway, MVPA levels could be significantly increased by exchanging driving for active transport such as walking or bicycling.

However, being physically active is not the only reason to participate in sport. In addition to hedonic pleasure, social interaction, and strength building, sport may provide significantly more vigorous PA than activities of daily living such as transportation. In our cohort, relative contributions to daily VPA from sport activities (leisure sport and PE) were larger (32%) than contributions from transportation (6%). Thus even if children receive large amounts of MVPA from non-sport domains such as transportation, leisure sport has many other benefits which should be appreciated.

### Study Strengths and Limitations

This large study (N>1,000) was done in Germany as part of the 15-year followup of two birth cohorts. We consider our study to be population-based, but for several reasons not perfectly representative of either Germany or Europe. Firstly, we included only newborns of German-speaking parents from two arbitrarily selected regions (see selection criteria for GINIplus and LISAplus.)[22] Secondly, while the primary response during recruitment was equally distributed across all social strata, the later dropout rate was higher for low-SES children, and consent for accelerometry was further dependent on SES: however, we find only weak evidence for selection for leisure sport participation within the cohort, and none for PE. Thus this study samples a relatively privileged, prosperous subset of the German population, likely even more selected than other populations where access to active transport or sport may be limited. Thus our estimates of the contributions of activity domains are likely to be population-specific.

Accelerometry is considered to be an objective form of PA assessment, and it is among the most objective under field conditions. However, the use of self-report (diary) data has almost certainly introduced some error into our PA estimates; and while we attempt to minimize this error as far as possible through scrupulous data cleaning, some almost certainly remains. A major concern of PA measurement is related to compliance of study participants, particularly in respect to keeping an adequate diary of device wear. [44] [45] This was the single biggest reason for a day to be invalid in our study, accounting for nearly 10% (1140/11,523) of recorded days: other reasons, such as technical issues, were comparatively minor. Ultimately 84% of subjects who completed accelerometry provided valid data, comparable to or slightly better than other large studies done in Europe [9] and Canada. [45] This dropout certainly introduced selection bias, which is most clearly visible in Table 1: accelerometry completers were likelier to be female and come from well-educated and urban families and thus may be more compliant; they may also be healthier and /or more health- and sport-conscious than the general population, and we may overestimate both participation and engagement in sporting activities even within the GINIplus and LISAplus cohorts.

Likewise, in order to reduce completion bias as much as possible we kept the diary relatively simple rather than breaking down the domains further, although this cost some precision. We were not able to estimate activity that took place outside of school, PE, transport to school, and leisure sport although this domain (“other time”) made up a large fraction of both total time and MVPA, including the trip home from school on every school day. Because many subjects did not go straight home, we were only able to profile the trip to school; and because subjects did not always diary the mode of transport to school we were not able to distinguish active transportation (walking, cycling) from passive transportation such as public transit or car. Lastly, our use of diary data to assign activity to the different domains has almost certainly introduced some error. We attribute 50% of MVPA to“other time,” but it is possible that some sporting activities took place during that time and were not diaried. However, selection is inevitable given the effort involved in participation and we consider this bias to be a reasonable price to pay for the high quality of data.

Accelerometry also has its own biases. Specifically it overmonitors high-acceleration activities such as trampolining, and undermonitors low-acceleration activities such as ice skating and cycling, relative to other measures of MVPA. [40, 46–49] It is not clear whether this is likely to produce an over- or underestimate of total MPA and VPA, but allocation of activity by domain may be biased towards those domains with significant levels of ambulation, and away from those domains with low ambulation but high MVPA, such as transportation to school if the student commutes by bicycle.

Epoch length (time over which accelerometric counts are summed) is also not yet standardized. We chose 60 seconds, but other studies of young people [11, 50] have used epochs as short as 5 seconds although 60 is the commonest. Shorter epochs often give higher estimates of moderate, vigorous, and MVPA than longer epochs, although differences are smaller in adolescents than in young children. Edwardson et al [50] found that when epoch length was increased from 5 to 60 seconds, moderate activity was not affected and vigorous activity declined by about 40%, for a slight (10%) decrease in MVPA. Guinhoya et al [11] concurred that the effects of epoch length on PA estimates were stronger for high activity levels than for low ones. This suggests that a shorter epoch length would have given higher estimates of vigorous activity but comparable ones for moderate and MVPA. However, since cardiovascular adaptation to PA is known to take several minutes [32] 60-second epochs may be physiologically the most relevant. 60-second epochs are also most intercomparable with other studies: both recent reviews [11, 51] found that 60 seconds was the most common epoch length. Thus we chose 60 seconds to maximize clinical plausibility and comparability with other studies, but recognize that other lengths are also defensible.

## Conclusions

These German adolescents were comparable to other Europeans in showing low levels of physical activity, with significant contributions from leisure sport and transportation to school. Leisure sport was the most active part of the day and contributed significant amounts of VPA, and participation was comparable between girls and boys and between the two study centers. Although most children had leisure sport, school PE, or both, transportation to school was frequent and thus accounted for a significant fraction of total MVPA. This indicates that even in a population with good access to dedicated sporting activities, frequent active transportation can add significantly to total MVPA.
